# Effectiveness of the socioecological informed contextual treatment summary and care plan (TSSCP-P, Brazil) for breast cancer survivors: a randomized, controlled study

**DOI:** 10.1007/s00520-024-08555-7

**Published:** 2024-05-23

**Authors:** Maria das Graças Silva Matsubara, Cristiane Decat Bergerot, Kimlin Tam Ashing, Fabiana Baroni Alves Makdissi, Simone Elias, Edvane Birelo Lopes De Domenico

**Affiliations:** 1 A.C.Camargo Cancer Center, Antônio Prudente Road, 211, São Paulo, SP 01509-010 Brazil; 2 Oncoclinicas&Co - Medica Scientia Innovation Research (MEDSIR), São Paulo, SP Brazil; 3 City of Hope Comprehensive Cancer Center, Duarte, LA USA; 4 A.C.Camargo Cancer Center, São Paulo, SP Brazil; 5 Universidade Federal de São Paulo, São Paulo, SP Brazil

**Keywords:** Breast cancer, Cancer survivors, Oncology, Patient care planning, Quality of life, Sickness impact profile

## Abstract

**Objective:**

This study aimed to evaluate the impact of an intervention using the Treatment Summary and Survivorship Care Plan (TSSCP-P) on self-efficacy and quality of life (QoL) in breast cancer survivors.

**Method:**

A clinical study, randomized and controlled, conducted to assess self-efficacy and QOL in breast cancer survivors. The participants were randomly assigned to either an intervention group or a control group. The intervention group received individualized nursing consultations guided by the TSSCP-P, while the control group received standard care. Self-efficacy and QoL were assessed at three evaluation moments using validated scales: *Functional Assessment of Cancer Therapy-Breast Plus Anm Morbidity* (FACT B + 4) and Perceived General Self-Efficacy Scale. Statistical analyses, including regression analysis and hypothesis tests, were conducted to examine the effects of the intervention on self-efficacy and QoL.

**Results:**

Female breast cancer survivors (*N* = 101) were recruited. The intervention group demonstrated a significant improvement in self-efficacy over time compared to the control group (*p* = 0.01). However, no significant differences were observed in the overall QoL scores between the two groups. Subscale analysis revealed a significant improvement in physical well-being for the intervention group (*p* = 0.04), while emotional well-being showed a non-significant improvement (*p* = 0.07). The study suggests that individualized care plans and support strategies, such as the TSSCP-P, can positively influence breast cancer survivors’ self-efficacy and certain aspects of their QoL.

**Conclusion:**

These findings highlight the potential benefits of the TSSCP-P intervention in enhancing self-efficacy among breast cancer survivors. However, further research is needed to explore its impact on overall QoL and its effectiveness across different stages of breast cancer, as well as with longer follow-up periods.

**Clinical trial registration number:**

Brazilian Registry of Clinical Trials (ReBEC- RBR-2m7qkjy; UTN code: U1111-1257–3560), registered in April 19th, 2022.

## Introduction

Breast cancer is the most common cancer among women worldwide, with 2.3 million diagnoses in 2020, resulting in 7.8 million women surviving in the last 5 years, making it the most prevalent globally [1, 2]. These survivors often experience late effects caused by cancer and its treatment, which have a negative impact on their quality of life (QoL) across multiple domains. Many needs of breast cancer survivors, such as information about treatment and its consequences, emotional support, and the importance of lifestyle changes, are not adequately met [3].

The benefits of suitable support, considering the specific biological, sociocultural, and emotional aspects of each survivor, were observed in studies that evaluated QoL and well-being, based on educational interventions for the development of self-management skills [4, 5]. The development of self-management depends on a favorable perception of self-efficacy, enabling individuals to make informed and autonomous decisions, such as actively seeking solutions to health problems, attending follow-up appointments, adhering to medication treatment, or adopting care measures [4, 6].

Therefore, a multimodal approach is necessary, incorporating supportive interventions with broad objectives, multidimensional and patient-centered care, which can be achieved with the implementation of individualized care plans for cancer survival, to the detriment of the practice of recent years, which consists of follow-up consultations focused only on clinical examinations and biological surveillance tests which results have already been shown to be inadequate, according to scientific literature [4, 5, 7, 8].

Since 2012, various North American organizations, including the Institute of Medicine (now Academies of Sciences, Engineering, and Medicine—NASEM), the Commission on Cancer, the LIVESTRONG Foundation, and the American Society of Clinical Oncology (ASCO), have advocated for the use of a Survivorship care plan (SCP) to improve the quality of care effectively. The SCP includes a treatment summary (TS) that provides pertinent information about the patient’s specific treatment, including clinical and surgical procedures, as well as details about the oncological disease and potential late or long-term treatment effects. Additionally, the SCP includes guidelines for alertness and health-promoting follow-up care [9–12].

Since then, several SCP models have been developed. One of these models, the Treatment Summary and Survivorship Care Plan (TSSCP) ASCO, was made available by ASCO in 2007 [12]. The TSSCP-ASCO model was subsequently translated into Spanish and adapted for use with Latin American women with breast cancer, based on the Shared Care and Psycho-Oncology Models, as well as the Contextual QoL [13]. This adaptation, known as the TSSCP-S version, underwent validation and demonstrated improved results compared to the original version in terms of content, clarity, cultural, linguistic, and socioecological responsiveness [13].

Based on the promising results of the TSSCP-S and the understanding that care plans need to be adapted to meet the linguistic and cultural needs of survivors for effective outcomes [14], a subsequent study was conducted to translate and validate the model in Brazilian Portuguese. This led to the development of the TSSCP-P, which demonstrated content validity indexes greater than 0.8 [15]. The TSSCP-P includes information on the definition and importance of a survivorship care plan, instructions on how to use it, breast cancer details, data related to cancer diagnosis and treatment, follow-up care and surveillance, clinical information, the care team, health advice, and questions about QoL [15].

Considering that the TSSCP-P is available for intervention studies and that there are currently no tested models in Brazil specifically designed to enhance the comprehensiveness and centrality of care for cancer survivors. The hypothesis of this study was that implementing a comprehensive survivorship care plan for women breast cancer survivors, including the TSSCP-P in the plan, would positively influence their QoL and perception of self-efficacy. Therefore, the main goal of this study was to measure the effectiveness of the TSSCP-P by assessing the QoL and self-efficacy of women who had survived breast cancer at three consecutive time points.

## Methods

### Study design and participants

An experimental, randomized study with a follow-up period ranging from two to twelve months, designed in accordance with the recommendations of CONSORT (Consolidated Standards of Reporting Trials) [16].

This study was conducted at a Cancer Center in São Paulo, SP, Brazil, and included patients who had completed treatment between January and August 2021. Data collection took place from June 2021 to May 2022. Recruitment was carried out through the electronic patient record (EPR), and the eligibility criteria were as follows: women over 18 years old, diagnosed with breast cancer in all pathological stages, who had completed surgical and clinical therapy (radiotherapy and antineoplastic chemotherapy, except endocrine therapy), and had follow-up consultations and/or outpatient care scheduled from June to September 2021. Patients who did not undergo surgical procedures for breast cancer treatment, had a history of cancers other than non-melanoma skin cancer, were not fluent in Portuguese, or had psychiatric disorders documented in the EPR that would hinder educational practices and men were excluded.

### Procedure

#### Data collect

After the approval from the respective Research Ethics Committees of the *Universidade Federal de São Paulo* and *Antônio Prudente Foundation* (protocol numbers 3,203,556/2019 and 3,351,638/2019), the eligible patients were recruited through the electronic patient record (EPR) after completion of treatment (except endocrine therapy). All participants were informed about the research objectives and type of participation desired. Anonymity and confidentiality were also assured, with the right to withdraw consent at any time, in accordance with Brazilian legislation on research in human and social sciences. Anonymity and confidentiality were also assured through the following procedures: medical records data were collected by two authorized researchers by the institution. They had access to the EPR and attributed an identification code for each participant. The data collected of each participant was anonymized and included by the same researchers in the institutional version of the REDCap® platform (Research Electronic Data Capture).

The eligible patients were contacted by telephone and upon acceptance were randomized based on their cancer staging and pre- and post-menopausal status, as these conditions could potentially affect the QoL of breast cancer survivors. The participants were assigned to different study arms through a completely random process, through software developed by the A.C.Camargo Cancer Center, for this purpose (https://accamargo.shinyapps.io/alocacao/), resulting in two groups: the experimental group (EG) and the control group (CG).

All interactions and data collection took place in person on the days that the participants had scheduled appointments (consultation and/or exams). At T1, the patients from EG and EC signed the consent form and completed sociodemographic and clinical questionnaires, as well as the General Perceived Self-efficacy and QoL scales (FACT-B + 4), which were also completed at times 1, 2, and 3.

#### Intervention

The interaction with all patients took place before or after outpatient care (medical consultation and/or examinations) and lasted between 30 and 90 min for EG, with longer time in T1 and 20 to 30 min for CG. The study consisted of three stages: pre-intervention (T1: *M* = 94.8 days, SD = 28.5), after the completion of surgical and clinical treatment (except endocrine therapy) (T2: *M* = 94.1 days after T1, SD = 23.6), and follow-up (T3: *M* = 91.0 days after T2, SD = 21.5). The selection of these time points was based on the follow-up protocol established for cancer survivors at the research institution, taking into consideration patient demand.

Patients randomized to the CG received the usual care offered by the institution, which involved a medical consultation to address any concerns related to the late side effects of treatments.

The educational intervention was conducted for EG participants at T1 by the main researcher and trained nurses, following a pre-prepared intervention plan to ensure a consistent approach. The intervention plan involved a specific and individualized approach to the participating women based on the components of the TSSCP-P, consisting of providing personalized information about possible late and long-term effects, strategies for management, identification of concerns, and development of action plans and goals for care and well-being. At that time, also an explanatory video about the plan was shown, and a printed copy of the TSSCP-P was provided. Participants completed the aforementioned instruments. At T2 and T3, participants had their questions clarified, which supported the development of new skills to cope with late effects. In T3, at the end of the application of the instruments, participants in the experimental group were invited to answer a question about their satisfaction with the care intervention, which consisted of: what is their satisfaction with the care and the usefulness of the care plan (TSSCP -P)? The possible answers were dichotomous, satisfied and dissatisfied.

In order to respect ethical precepts, at the conclusion of the study, CG participants received at T3 the training and the printed TSSCP-P, along with the explanatory video that had been provided to the EG participants.

## Measures

Sociodemographic and clinical data were collected through the EPR and clinical interviews.

The assessment of self-efficacy was conducted using the General Perceived Self-Efficacy Scale, which was designed to measure a general sense of perceived self-efficacy, with a specific focus on adaptation after experiencing a stressful event. The respondents were instructed to read the sentences and select the number that best described themselves, based on a four-point Likert-type scale ranging from 1 (not true) to 4 (completely true). The total score ranges from 10 to 40, with higher values indicating a greater perception of self-efficacy [17].

To evaluate the QoL, the Functional Assessment of Cancer Therapy-Breast plus Arm Morbidity (FACT-B + 4) questionnaire was utilized. This questionnaire is recommended for assessing the QoL of breast cancer patients and has demonstrated superior results compared to other instruments [18]. The scale consists of 41 questions, with 27 items assessing general QoL (FACT-G) and covering domains such as physical well-being (seven items), social/family well-being (seven items), emotional well-being (six items), and functional well-being (seven items). Additionally, there are ten items specifically addressing breast cancer-related problems (FACT-B) and four supplementary questions assessing arm morbidity in patients who underwent surgery for breast cancer (FACT-B + 4). All items are rated on a five-point Likert-type scale, where higher scores indicate better QoL. The total score can range from 0 to 164 [18].

## Statistical analysis

Recruitment criteria were established based on regression analysis. For the calculation, we estimated a minimum of 20 cases per variable (self-efficacy and QoL), resulting in a total of 40 participants. Additionally, we incorporated an extra contingency to accommodate potential losses [19]. In all hypothesis tests, a significance level of 5% was adopted. Therefore, results with *p* values less than 0.05 were considered statistically significant. Data analysis was performed using the free software R version 3.5 and IBM SPSS software version 25.

Descriptive analyses, including frequencies, percentages, means, standard deviations (SD), and intervals, were employed to characterize the patients. The independence test, Chi-square test, and Fisher’s exact test were used to compare clinical and sociodemographic variables between the groups. To evaluate the effect of the intervention over time, the analysis of variance (ANOVA)-Repeated Measures test was utilized, specifically applied to the self-efficacy and FACT-B + 4 scales at the three evaluation points for both the control and experimental groups. This technique facilitated the identification of significant differences between the groups and/or evaluation points, while accounting for the correlation between repeated measures. The variance–covariance matrix was examined to ensure the accuracy of the results and detect any significant effects of the intervention over time. Furthermore, age, marital status, and education were included as covariates in the statistical analysis.

## Results

**Fig. 1:**
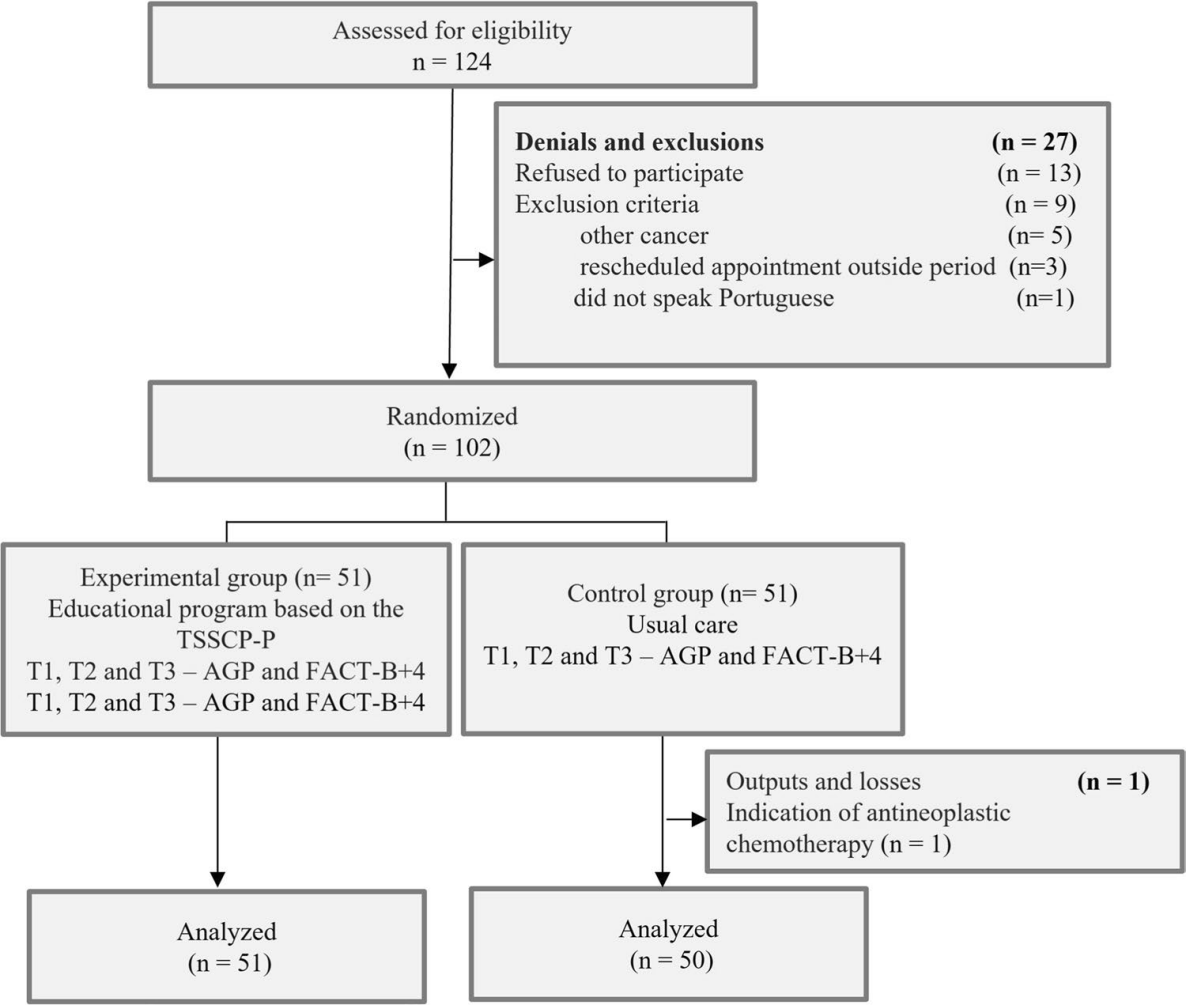
CONSORT 2010 flowchart. *Note. AGP* General Perceived Self-Efficacy, *FACT-B* + *4* Functional Assessment of Cancer Therapy-Breast plus Arm Morbidity

At the time of recruitment, 124 patients who met the eligibility criteria had appointments scheduled 2 to 6 months after the completion of surgical and clinical treatment (except endocrine therapy), as shown in Fig. 1.

## Characteristics of the participants

The characteristics of the patients were well balanced, with no significant differences identified between the groups, except for lymphedema (Table 1). Table 1 presents the clinical and sociodemographic data.

**Table 1:** Comparison of sociodemographic and clinical characteristics of women who survived breast cancer in the experimental and control groups

Sociodemographic characteristics	EG (*N* = 51)	CG (*N* = 50)	Total	*p* value
Age [*M* (SD)]				
	55.7 (12.1)	52.7 (12.4)	54.2 (12.3)	0.22
Marital status [*N* (%)]				0.53
Married/stable union	66.7 (34)	64.0 (32)	65.3 (66)	
Single	13.7 (7)	22.0 (11)	17.8 (18)	
Widow	9.8 (5)	10.0 (5)	9.9 (10)	
Divorced	9.8 (5)	4.0 (2)	6.9 (7)	
Level of education [*N* (%)]				0.83
Superior	72.5 (37)	78.0 (39)	75.2 (76)	
High school	19.6 (10)	14.0 (7)	16.8 (17)	
Elementary	7.8 (4)	8.0 (4)	7.9 (8)	
Income [*N* (%)]				0.72
High income	31.4 (16)	22.0 (11)	26.7 (27)	
Middle income	60.8 (31)	66.0 (33)	63.4 (64)	
Low income	7.8 (4)	12.0 (6)	9.9 (10)	
Spirituality [*N* (%)]				0.83
Catholicism	58.8 (30)	58.0 (29)	58.4 (59)	
Evangelical	15.7 (8)	12.0 (6)	13.9 (14)	
Spiritism	13.7 (7)	10.0 (5)	11.9 (12)	
Does not have	2.0 (1)	2.0 (1)	3.0 (3)	
Others	9.8 (5)	16.0 (8)	12.8 (13)	
Comorbidity [*N* (%)]				0.43
Yes	64.7 (33)	74.0 (37)	69.3 (70)	
Uses endocrine therapy [*N* (%)]				0.31
Yes	78.4 (40)	88.0 (44)	83.2 (84)	
Type of therapy used [*N* (%)]				0.98
Aromatase inhibitor	55.0 (22)	52.3 (23)	53.6 (45)	
Tamoxifen	45.0 (18)	47.7 (21)	46.4 (39)	
Histology [*N* (%)]				
Ductal carcinoma in situ	23.5 (12)	24.0 (12)	23.8 (24)	1.00
Non-special type ductal carcinoma	72.5 (37)	64.0 (32)	68.3 (69)	0.48
Invasive lobular carcinoma	9.8 (5)	12.0 (6)	10.9 (11)	0.97
Molecular subtype [*N* (%)]				
Luminal A	33.3 (17)	32.0 (16)	32.7 (33)	1.00
Luminal B	45.1 (47)	48.0 (24)	46.5 (47)	0.93
Triple negative	7.8 (4)	4.0 (2)	5.9 (6)	0.68
HER2 overexpression	0.0 (0)	6.0 (3)	3.0 (3)	0.12
Pathological staging [*N* (%)]				0.78
0	15.7 (8)	22.0 (11)	18.8 (19)	
I	33.3 (17)	28.0 (14)	30.7 (31)	
II	33.3 (17)	36.0 (18)	34.7 (35)	
III	15.7 (8)	10.0 (5)	12.9 (13)	
Treatment [*N* (%)]				0.82
Surgery	7.8 (4)	10.2 (5)	9.0 (9)	
Surgery + antineoplastic chemotherapy	5.9 (3)	4.1 (2)	5.0 (5)	
Surgery + radiotherapy	52.9 (27)	44.9 (22)	49.0 (49)	
Surgery + antineoplastic chemotherapy + radiotherapy	33.3 (17)	40.8 (20)	37.0 (37)	
Presence of lymphedema [*N* (%)]				**0.05**
No	90.2 (46)	100.0 (50)	95.0 (96)	

^*Note*. Statistically significant difference for*p* ≤0.05; there was a patient with more than one histological type of breast cancer^

^Abbreviations.*p*statistical significance value^

## Intervention effect

**Fig. 2:**
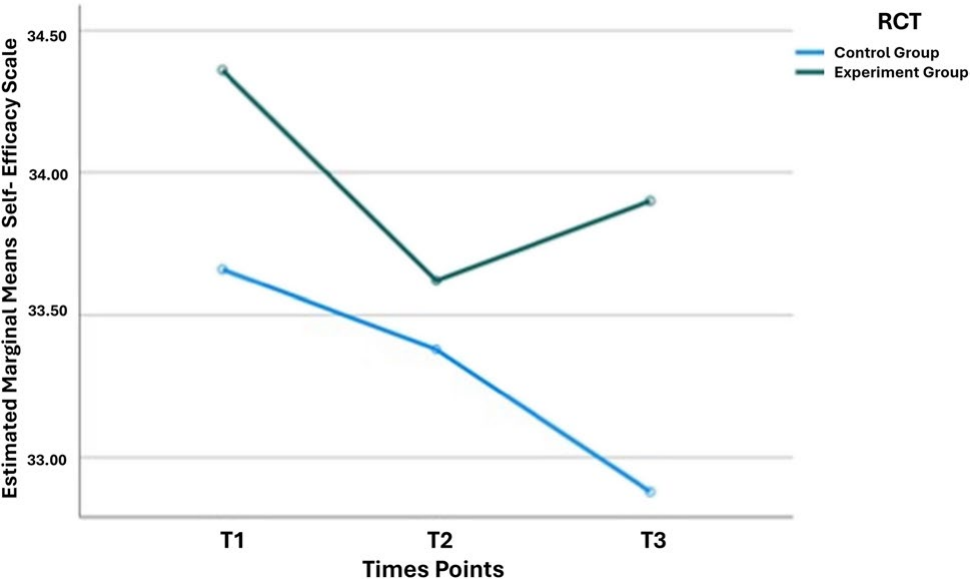
Line graph indicating the total self-efficacy scores, from baseline to T3 (12 months), with improvement in results reported by the EG (*p* = 0.01)

The patients’ reported self-efficacy indicated an improvement in the results reported by the experimental group (EG). The assumption of sphericity, as assessed by Mauchly’s test, was met, and a significant main effect over time was found for the self-efficacy scale (Table 2). Additionally, a combined effect of the group type and self-efficacy over time was observed (*p* = 0.01) (Fig. 2).

**Table 2:** Self-efficacy and QoL reported by participants in the experimental and control groups, in the three assessment times

	T1		T2		T3		
Variables	EG (*N* = 51)	CG (*N* = 50)	GE (*N* = 51)	GC (*N* = 50)	GE (*N* = 50)	GC (*N* = 50)	*p* value
Self-efficacy scale [M]	34.4	33.6	33.6	33.3	33.9	32.8	0.04
FACT-B + 4 scale [M]	103.7	102.2	100.6	101.8	101.5	102.3	0.11
Physical well-being subscale	22.3	21.9	20.8	21.7	22.0	21.8	0.04
Emotional well-being subscale	20.4	19.6	19.1	19.5	19.6	19.5	0.07

T1: (M = 94.8 days, SD = 28.5), after the completion of surgical and clinical treatment (except endocrine therapy), T2: (M = 94.1 days after T1, SD = 23.6), and follow−up, T3: (M = 91.0 days after T2, SD = 21.5)

*Note*. Statistically significant difference for p ≤ 0.05; there was a patient with more than one histological type of breast cancer

Abbreviation. *p,* statistical significance value

**Fig. 3:**
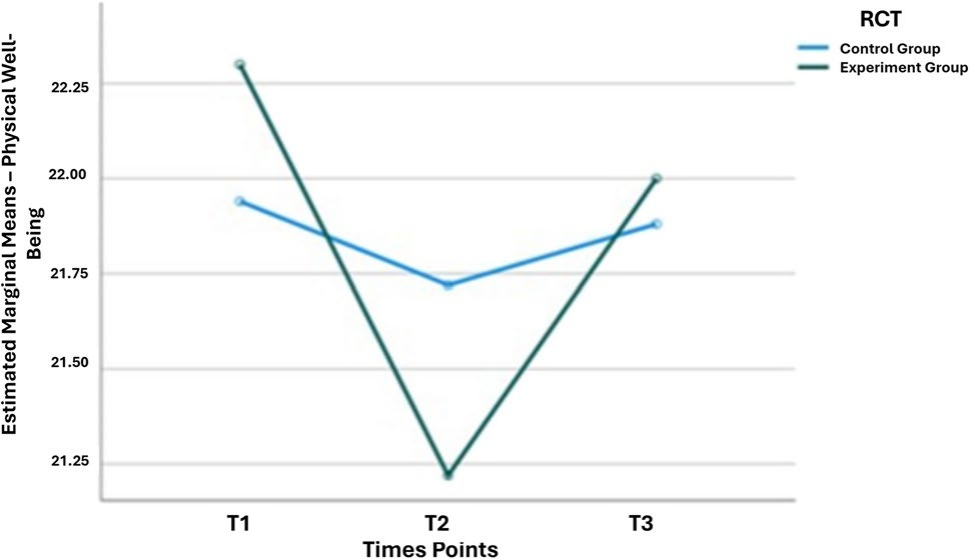
Line graph indicating the total scores of the physical well-being subscale, from baseline to T3 (12 months), with improvement in results reported by the EG (*p* = 0.04)

However, in the physical well-being subscale, a significant effect over time was observed, with better results reported by the EG (Fig. 3). In the emotional well-being subscale, there was a non-significant improvement over time, favoring the EG (Table 2). Regarding the FACT-B + 4 scale, no significant changes were identified over time in either the GC or the EG (Table 2).

The data obtained from EG participants regarding satisfaction with the care intervention totaled 90.4% positive responses.

## Discussion

In this study, we evaluated the impact of an intervention developed from the application of the TSSCP-P on self-efficacy and QoL for breast cancer survivors, and our results showed patient satisfaction, corroborating similar studies [20, 21].

We observed a significant improvement in self-efficacy over time in the intervention arm. Self-efficacy is an important factor in improving healthcare outcomes for cancer survivors, being able to reduce the severity of symptoms and self-care behaviors, as individuals’ beliefs in their ability to achieve goals and their commitment to meeting them are influenced by their perceived efficacy [22–25]. However, our result differs from a meta-analysis of eight studies that compared the effects of care plans for cancer survivors, which found no significant differences in self-efficacy between groups over time [22]. This difference can be explained by the fact that increased self-efficacy is associated with support strategies for self-management, such as increased knowledge and skill development, which in turn impact patient confidence, in managing the physical and psychosocial effects [26]. Therefore, structured and individualized tools based on cognitive, affective, and social components can facilitate behavior change [27].

A study comparing self-efficacy between cancer patients and the general population, with a sample of 959 participants, demonstrated significant results for all dimensions of the Core QoL (EORTC QLQ-C30) (*p* < 0.001), with higher coefficients for emotional functioning [28]. The results showed that patients with higher self-efficacy had better QoL outcomes [28]. Similar findings were observed in our study, with a significant improvement in physical well-being and a tendency towards improvement in emotional well-being, which converges with the use of the care plan in other countries, as shown in a systematic review that aimed to evaluate the use and impact of SPC on breast cancer survivors, showing improvements in survivorship care knowledge and emotional and physical well-being [21].

The emphasis on engagement and the use of approaches focused on supporting self-care and self-management, including information on managing physical side effects, addressing doubts, and developing new coping skills, may have contributed to the impact demonstrated in our study in terms of emotional and physical aspects. Additionally, health education with detailed information and interactive strategies for communication can improve symptom understanding, providing patients with tranquility and confidence [29–31].

However, we did not observe a significant difference between groups over time in the global QoL score. This result aligns with the findings of two systematic reviews (*N* = 2286 and *N* = 3798) that evaluated the effects of care plans on QoL in cancer survivors, which showed no sustained effects on QoL but a reduction in unmet needs after implementation [21, 32]. It should be noted that breast cancer survivors are at high risk of experiencing physical, functional, emotional, and psychosocial changes that can significantly impact their daily lives [31]. However, despite an overall tendency for QoL to improve over time, adverse effects related to therapies may persist for longer than 6 months [33, 34].

Considering the focus of our study on breast cancer survivors in the early post-treatment months, it is expected that patients will report treatment-related side effects during this initial phase. Moreover, these symptoms can influence various domains of QoL. This was demonstrated in an experimental longitudinal study (*N* = 202 women who survived breast cancer) that evaluated the effect of a care plan delivered through a mobile app (eHealth) on QoL. The findings indicated a significant increase in QoL scores (*p* = 0.000) in the intervention group after 1 year of follow-up [29]. Significant effects on emotional well-being were observed at the sixth month; however, these effects were not sustained at the 1-year follow-up [29]. For many women, this transition period is challenging as they face unexpected physical and emotional effects, which can disrupt their expectations of returning to life before the cancer diagnosis [35]. Therefore, it is essential to identify these needs and provide timely and accessible support for this population, utilizing individualized care plans as a tool [35].

It is important to highlight that the process of adapting to breast cancer depends on the characteristics of the individual woman, her clinical situation, and how she confronts the specific challenges of each phase of the disease. These unique factors can significantly influence both the QoL and the adoption of behaviors that minimize risk factors [34]. Many female breast cancer survivors report a lack of comprehensive and up-to-date information to manage side effects after treatment [36]. In this context, the TSSCP-P can be an essential tool as it provides information that aligns with the needs of the studied population and focuses on developing skills to navigate the initial post-treatment survival phase. However, it should not be limited to the mere delivery of a document but rather incorporated into a comprehensive care plan for survivorship [37]. In our investigation, participants in the intervention group underwent three well-planned nursing consultations that took an individualized approach, with the TSSCP-P serving as a guide for their care.

Thus, we found that the follow-up assessment of women who have survived breast cancer is a complex and multidimensional process that goes beyond checking clinical, laboratory, and imaging tests solely for the purpose of recurrence surveillance, which is an inevitable concern. The care of breast cancer survivors can benefit from the delivery and monitoring of the ongoing activities outlined in the TSSCP-P, not only for the outcomes demonstrated in our study but also for its potential to enhance self-knowledge, self-management, awareness of inherent risks in the survivorship phase, and encourage patients to promptly report symptoms.

## Study limitations

This study has several limitations that should be taken into account when interpreting the results and drawing conclusions. Firstly, the sample size was restricted and limited to a single private institution, which may limit the generalizability of the findings. Additionally, the assessment was conducted over a relatively short period of time, potentially limiting the ability to capture long-term changes. In addition, an important consideration is that the sample consisted of breast cancer survivors with a sociodemographic profile that may differ from the broader Brazilian population, particularly in terms of lower educational levels and socioeconomic conditions. This could impact the results and hinder generalization to other populations. Another limitation of this study is the reliance on self-reported measures, which may introduce response bias and affect the accuracy of the data collected. Lastly, conducting the study during the pandemic period may have influenced the QoL outcomes, as some patients reported experiencing grief, illness among friends and family members due to COVID-19, or even post-infection sequelae.

## Clinical implications

The clinical implications of this study are particularly relevant for developing countries, where breast cancer survivors often encounter challenges related to limited medical care and issues of access and quality. The findings suggest that the use of the TSSCP-P can offer benefits in addressing these challenges. However, further research is needed to assess the effectiveness of the TSSCP-P in various stages of breast cancer and with longer follow-up periods. This would provide a more comprehensive understanding of which participants may derive the greatest benefits from this intervention.

## Conclusion

The study findings suggest that the intervention using the TSSCP-P had a positive impact on the self-efficacy of breast cancer survivors. The participants reported improvements in their belief and confidence in managing their health and well-being over time. However, no significant changes were observed in the overall QoL scores, although there was a significant improvement in physical well-being. The study highlights the importance of individualized care plans and support strategies, such as the TSSCP-P, in addressing the unique needs and challenges faced by breast cancer survivors during their post-treatment phase. Further research is warranted to explore the effectiveness of the intervention across different stages of breast cancer and with longer follow-up periods, to better understand its potential benefits and impact on QoL outcomes.

## Data Availability

The data that support the findings of this study are available on request from the corresponding author. The data are not publicly available due to privacy or ethical restrictions.
